# Autologous bone marrow stem cell intralesional transplantation repairing bisphosphonate related osteonecrosis of the jaw

**DOI:** 10.1186/1746-160X-7-16

**Published:** 2011-08-17

**Authors:** Luigi Cella, Aldo Oppici, Mariacristina Arbasi, Mauro Moretto, Massimo Piepoli, Daniele Vallisa, Adriano Zangrandi, Camilla Di Nunzio, Luigi Cavanna

**Affiliations:** 1 Departments of Oral and Maxillofacial Surgery, Hospital of Piacenza, Via Taverna, 49. 29100. Italy; 2 Department of Immunohematology, Hospital of Piacenza, Via Taverna, 49. 29100. Italy; 3 Department of Cardiology, Hospital of Piacenza, Via Taverna, 49. 29100. Italy; 4 Department of Oncology and Hematology, Hospital of Piacenza, Via Taverna, 49. 29100. Italy; 5 Department of Pathology, Hospital of Piacenza, Via Taverna, 49. 29100. Italy

**Keywords:** Osteonecrosis of the Jaw, bisphoshonate, stem cell transplantation, organ repair

## Abstract

**Purpose:**

Bisphosphonate - related osteonecrosis of the JAW (BRONJ) is a well known side effect of bisphosphonate therapies in oncologic and non oncologic patients. Since to date no definitive consensus has been reached on the treatment of BRONJ, novel strategies for the prevention, risk reduction and treatment need to be developed. We report a 75 year old woman with stage 3 BRONJ secondary to alendronate and pamidronate treatment of osteoporosis. The patient was unresponsive to recommended treatment of the disease, and her BRONJ was worsening. Since bone marrow stem cells are know as being multipotent and exhibit the potential for differentiation into different cells/tissue lineages, including cartilage, bone and other tissue, we performed autologous bone marrow stem cell transplantation into the BRONJ lesion of the patient.

**Methods:**

Under local anesthesia a volume of 75 ml of bone marrow were harvested from the posterior superior iliac crest by aspiration into heparinized siringes. The cell suspension was concentrated, using Ficoll - Hypaque^® ^centrifugation procedures, in a final volume of 6 ml. Before the injection of stem cells into the osteonecrosis, the patient underwent surgical toilet, local anesthesia was done and spongostan was applied as a carrier of stem cells suspension in the bone cavity, then 4 ml of stem cells suspension and 1 ml of patient's activated platelet-rich plasma were injected in the lesion of BRONJ.

**Results:**

A week later the residual spongostan was removed and two weeks later resolution of symptoms was obtained. Then the lesion improved with progressive superficialization of the mucosal layer and CT scan, performed 15 months later, shows improvement also of bone via concentric ossification: so complete healing of BRONJ (stage 0) was obtained in our patient, and 30 months later the patient is well and without signs of BRONJ.

**Conclusion:**

To our knowledge this is the first case of BRONJ successfully treated with autologous stem cells transplantation with a complete response.

## Background

Bisphosphonates are widely used in the management of bone diseases including osteoporosis, Paget's disease, hypercalcemia related to malignancy and in the prevention of skeletal complication from bone metastasis. Bisphosphonates are incorporated into skeleton and suppress bone resorption, without being degraded [1,2]. Bisphosphonates have shown direct anti-tumor effects, possibly related to growth factors release reduction and inhibition of cell adhesion molecules [2,3]. Bisphosphonates - related osteonecrosis of the Jaw (BRONJ) has been characterized as a main side effect of bisphosphonate therapy [4-6]. The most frequent clinical sign of BRONJ is bone exposure, associated with pain, swelling and purulent secretion that does not heal over a period of 6-8 weeks [7,8]. To date no definitive consensus has been reached on the treatment of BRONJ and several studies reported relatively conflicting results following surgery, antibiotics, laser or hyperbaric oxygen administration [9-16]. For this reason, new strategies for the prevention, risk reduction and treatment for BRONJ need to be developed [16,14-20].

Bone marrow harvested stem cells and progenitor cells (BMSC) may be capable of solid-organ repair [21], and it has been demonstrated that adult human mesenchymal stem cells (MSC) from bone marrow can represent a promising source for skeletal regeneration [22].

Based on these data, we report here a patient with BRONJ, unresponsive to the recommended procedures, that showed clinical and radiographic improvement after autologous bone marrow stem cells intralesional transplantation.

## Case report

**Figure 1:**
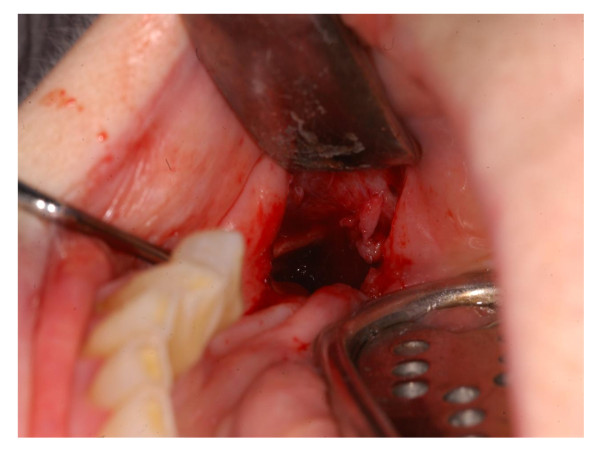
**Clinical onset and appearance of BRONJ stage III**.

In January 2008 a 75 year old woman was referred to us for a stage III BRONJ of the mandible (Figure 1); she was previously treated for a severe osteoporosis with alendronate 70 mg every four weeks for 9 months, then pamidronate 60 mg every four weeks for two years. In the same period the patient was treated with Eritropoietin beta (EPO) for three years (10.000 U/weeks) for a mild renal failure related anemia. BRONJ was defined in accordance with the American Association of Oral and Maxillofacial Surgeons Position Paper on bisphosphonates - related osteonecrosis of the Jaws [23,17] and all the three characteristics were present in the patient:

1. Current or previous treatment with a bisphosphonate;

2. Exposed bone in the maxillofacial region that has persisted fore more 8 weeks;

3. No history of radiation therapy to the jaws.

**Figure 2:**
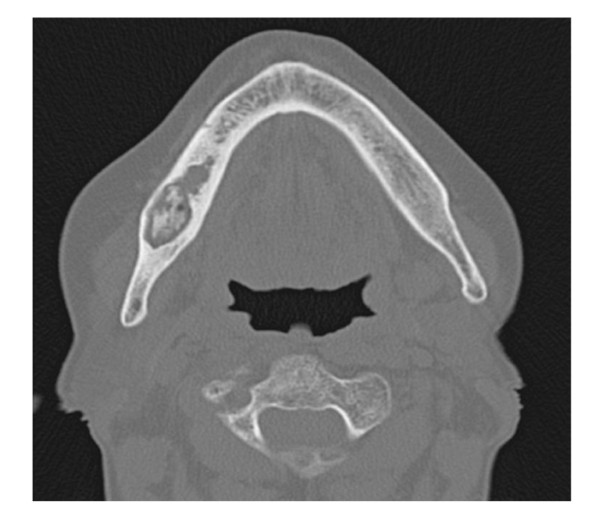
**Clinical Onset: computed tomography scan shows bone destruction**.

Conservative, non - surgical treatment was initially performed, as recommended [17-20], such as oral hygiene (brushing and mouth rinses), topical and systemic antibiotics active against common oral/dental bacterial infection; subsequently both debridement, toilet of exposed bone and Er:YAG laser treatment were uneffective [15,16] and patient's conditions deteriorated with pain and worsening of BRONJ progressively. Computed Tomography (CT) scan showed bone destruction (Figure 2). Resection and immediate reconstruction was proposed to the patient, however she refused resection, as recommended [17,20].

The concept that bone marrow stem cells upon transplantation into adult recipients transdifferentiate and contribute to the rigeneration of a variety of non -hematopoietic lineages in multiple organs, has provoked great interest for its potential clinical applications [24,25] as recently reported also by our group [26]. So we offered the opportunity to our patient of injection of autologous bone marrow stem cells into the osteonecrosis site lesion.

## Methods

In september 2008 the patient (who is the mother of one of us) was informed about the procedure and gave written informed consent.

Under local anesthesia an average of 75 ml of bone marrow were harvested from the posterior superior iliac crest by aspiration into heparinized syringes as previously reported by our group [26]. Progenitor cells were isolated and enriched using Ficoll - Hypaque^® ^centrifugation procedures. This procedure allowed the depletion of mature myeloid and erythroid cell from the harvest. The cell suspension consisted of an heterogeneous cell population including hematopoietic, mesenchymal, endothelial and other progenitor cells as well as mononuclear cells. The cells were suspended into an opportune quantity of PBS-EDTA buffer containing 5% of human albumin.

The cell fraction was concentrated in a final volume of 6 ml. Finally, before intralesional transplantation, the cells were subjected to quality control procedures (i.e. sterility test for aerobic and anaerobic bacteria, Elisa test for HCV, HBV, HIV viruses) in order to exclude any contamination as previously reported [26]. In addition, full blood count and immune-phenotype analyses of the cell suspension were performed, including absolute CD34 and CD45 positive cell count and five colour MoAb panel for the identification of the cellular subpopulation (Table 1).

**Table 1:** Multiparameter flow cytometric analysis of the injected BMC

Cellular subset	harvest (ml 75)	final (ml 6)	injected (ml 4)
nuclear cells/ul (total E^6^)	16.800/ul (1.260)	71.000/ul (426)	
WBC (CD45^+^) (E^6^) *	875,7	252	168
CD 34+ cells/ul	95,2	800	800
CD 34+ cells (E^6^)	7,14	4,8	3,2
CD34+/CD117+ cells (E^6^)	5,64	4,22	2,8
CD34+/CD133+ cells (E^6^)	3,42	1,15	0,76
CD45-/CD31+ cells (E^6^)	2,64	0,97	0,64
CD133+/CD117+ cells (E^6^)	4,1	1,95	1,3
CD133+ cells (E^6^)	5,92	2,25	1,5
CD117+ cells (E^6^)	28	20,2	13,5
CD45-/CD105+/CD71- cells (E^6^)	302,2	255,6	170
Ficoll mediated myeloid depletion % *			

BMC: Bone marrow cells

Before the injection of stem cells into the osteonecrosis the patient underwent a surgical toilet in local anesthesia of the bone lesion.

The bone cavity was fullfilled with fibrine sponge (Spongostan^®^) as a carrier, then 4 ml of stem cells suspension and 1 ml of patient's activated platelet-rich plasma were injected in the lesion of BRONJ.

## Results

**Figure 3:**
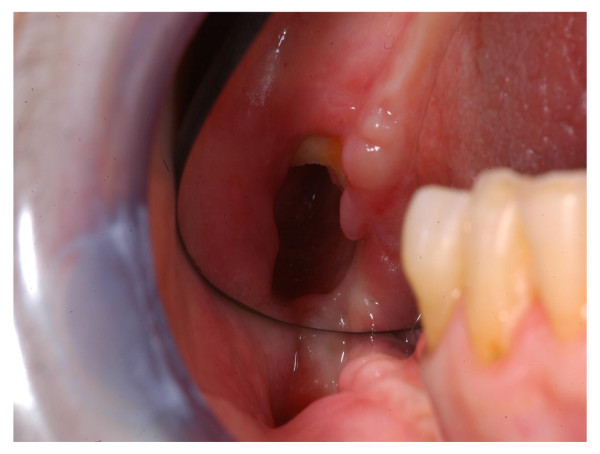
**Two weeks later after bone marrow cells transplantation: pink coloured new layer shows progressive improvement of the mucosa**.

**Figure 4:**
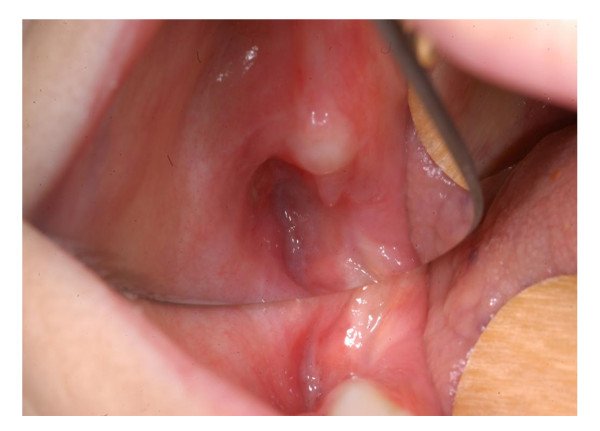
**Four months later: the lesion of the mucosa is ulteriorly improved**.

**Figure 5:**
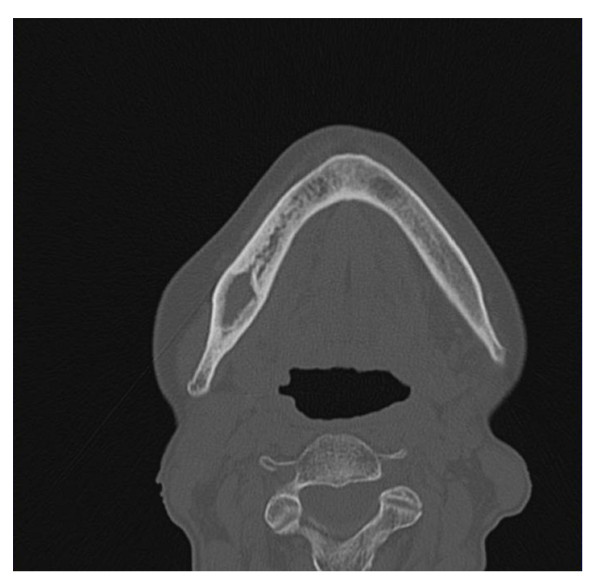
**Computed Tomography scan, 15 months later, shows a concentric ossification of the bone lesion**.

The procedure was well tolerated, and a week later the dehiscence of the surgical wound was observed, the residual carrier was removed. Then a soft, uniform layer of whitish mucosa dressing the bone cavity was observed. Two weeks later, resolution of symptoms was obtained and the lesion improved (Figure 3) with the pink coloured new layer. Subsequently the patient was seen at our out patients dental-maxillofacial service every two weeks for six months, then every four weeks and showed a progressive improvement. Clinical controls showed progressive improvement of the mucosal layer (Figure 4). CT scan performed 15 months later showed improvement of bone and concentric ossification (Figure 5). A complete healing of BRONJ (stage 0) was obtained and the patient is well without sings of BRONJ 30 months later. To our knowledge this is the first case of BRONJ treated with autologous stem cells injection.

## Discussion

Since the first description by Marx, 2003 [27]and Wang et al 2003 [28], cases with BRONJ are being increasingly reported, first of all, in oncologic patients in line with the increased use of bisphosphonates (mainly zolendronate and pamidronate) as the main pathogenetic factor of BRONJ.

A review of the literature through march 2006 performed by our group [9] identified more than 250 reported case on BRONJ, and more recently over 6.000 cases have been reported to the US Food and Drug Administration [29]. The treatment goals for patients with an established diagnosis of BRONJ are, as recently reported [16-20], to eliminate pain, to control infection of the soft and hard tissue and to minimize the occurrence or the progression of bone necrosis.

However the response to treatments of the patients with BRONJ is less predictable than the established surgical treatment modalities for osteomyelitis or osteoradionecrosis, and new treatment procedures need to be developed [16-20].

From the hystopathological point of view the BRONJ is characterized by an avascular necrosis. In osteonecrosis is found a lack of osteogenic precursor cells that derive from mesenchymal stem cells (MSCs), but also a lack in vascular support that derives from endothelial progenitor cells (EPCs).

MSCs are known as being multipotent and exhibit the potential for differentiation into different cell/tissue lineages, including cartilage, bone, adipose tissue, tendon and ligament [30]. These pluripotent mesenchymal progenitor cell are denoted as stromal or mesenchymal stem cells.

In vivo osteogenesis occurs only if the density of implanted cells at the treated site is sufficiently high. To achieve this goal, either large amounts of concentrated bone marrow stem cells or bone marrow stem cells in combination with growth factors can be used [31,32].

This situation has been reproduced by in vitro studies which confirmed that composite implantation of mesenchymal stem cells with endothelial progenitor cells enhances tissue-engineered bone formation [33].

The homing mechanisms of MSCs are poorly understood; it is known that, based on chemokine/chemokine-receptor interactions and adhesion molecules, MSCs are potentially capable on finding the site of injury and when, given intravenously, of restoring damaged tissue on site due to their plasticity and/or paracrine properties [30]. However, it must be emphasized that a direct approach, bringing direct into the osteonecrotic site a significant amount of bone marrow enriched in mononuclear cells, could allow a better osteogenesis of the damaged bone based on evidence data of the presence in this cell-fraction of osteoid and angiogenic precursors [34-36].

Bone marrow contains three main cell lines: hematopoietic cells, mesenchymal and proendotelial cells [34,35]. Recently we reported in a randomized controlled trial the effects of intracoronary transfer of autologous bone marrow stem cells in patients with acute anterior myocardial infarction and we demonstrated that this procedure improves cardiac, autonomic, and functional indexes in this setting of treated patients [26]. These positive effects may be mediated by a direct transdifferentiation of transplanted stem cells to cardiomyocytes [37], but also indirectly by parackrine secretion of cytokines and growth factors with resulting stimulation of survivors cardiac stem cells and/or angiogenesis, improving microvascular function [38]. Recently, a stabilizing effect of the injection cells via changes in the connettive tissue has been hypothesized [39].

Stem cells are easily obtained from the bone marrow with a minimally invasive approach and can be easily transplanted into the osteonecrotic lesion as demonstrated in our patient. This simple, cheap procedure allowed a clinical improvement of symptoms, and induced novel ossification as demonstrated by CT scan 15 months after the treatment, and it must be emphasized that the patient is in complete remission from a stage 3 BRONJ after 30 months. In addition, our patient showed a particularly rich bone marrow, not only in red cells precursors (as we expected since the patient was treated with EPO), but also in total stem cells subset (table 1). Recently, Kikuiri et al, [40] infused mesenchymal stem cells in BRONJ-like mice. They demonstrated that systemic infusion with MSCs prevents and cures BRONJ-like disease possibly via introduction of peripheral tolerance, shown as an inhibition of T-helper-producing interlukin 17 cells (th17)and increase in T regulatory cells (Tregs). Handschel and Meyer [41] suggest that stem cells might be a promising treatment option for BRONJ and our case demonstrates their hypothesis is right. In our case bone marrow stem cells were directly infused in the bone lesion of BRONJ with a complete remission.

We are aware that a case report can be of limited interest, however it could suggest that this technique may be studied in patients with BRONJ unrensponsive to standard treatment and can be tried before major demolitive surgery procedures for the reconstruction of defect of the ONJ by bisphosphonates.

## Consent

Written informed consent was obtained from the patient for publication of this case report and accompanying images. A copy of the written consent is available for review by the Editor-in-Chief of this journal

## Competing interests

The authors declare that they have no competing interests.

## Authors' contributions

All authors read and approved the final manuscript. LC, MA, LC conceived of the study, and participated in its design and coordination and helped to draft the manuscript. AO, MM have been involved in drafting the manuscript and to collect the results from follow-up examinations. MP has been involved in revising the manuscript critically for important intellectual content. DV, AZ, CDN have done substantial contributions to conceptions to conception and design and interpretation of data.
